# The Construction of a Mutant Library of Linseed and the Molecular Mechanism of High Oleic Acid Mutants from a Multi-Omics Perspective

**DOI:** 10.3390/plants14162583

**Published:** 2025-08-20

**Authors:** Caiyue Liu, Xinsen Yang, Qiaoling Tang, Xiuxia Cao, Aiping Qian, Zhiwei Yang, Limin Wang, Jianping Zhang, Xinwu Pei, Lu Gan

**Affiliations:** 1 Biotechnology Research Institute, Chinese Academy of Agricultural Sciences, Beijing 100081, China; caiyueliu@163.com (C.L.); xinsenyang@126.com (X.Y.); tangqiaoling@caas.cn (Q.T.); 2 Guyuan Branch of Ningxia Academy of Agriculture and Forestry Sciences, Guyuan 756000, China; kyglk@126.com (X.C.); gyqap@163.com (A.Q.); yzw425122541@163.com (Z.Y.); 3 Crop Institute, Gansu Academy of Agricultural Sciences, Lanzhou 730070, China; liminwang@aliyun.com (L.W.); zhangjpzw3@gsagr.ac.cn (J.Z.)

**Keywords:** high-oleic acid, flax, EMS, multi-omics

## Abstract

Flax (*Linum usitatissimum* L.) is a globally important oilseed crop, valued for its edible and industrial uses. Flax seeds are rich in unsaturated fatty acids. In this study, ethyl methyl sulfone was employed to construct a mutant library from the flax cultivar Longya 10 (WT). Screening efforts identified M45, a stable mutant with an oleic acid content of 43.22% at 40 days after flowering, representing a 21.23% increase over the wild-type. RNA-Seq analysis revealed the presence of two homologs of the *SAD* (stearoyl-ACP desaturase) family and two homologs of the *FAD2* (fatty acid desaturase 2) family, which showed differential expression in a trend consistent with the phenotype of M45. A BSA-Seq analysis was conducted to identify genes with SNPs (single nucleotide polymorphisms) and Indel (insertions/deletions) variant loci that were associated with increased oleic acid. The combination of BSA-Seq, RNA-Seq, and metabolomic analyses identified *L.us.o.g.scaffold122.86*, a gene that may be co-expressed with *L.us.o.g.scaffold7.26* to affect oleic acid accumulation via *FAD2*.

## 1. Introduction

Flax, or linseed (*Linum usitatissimum* L.), is a highly valued oilseed crop grown in more than 50 countries. It is used for food, animal feed, and industrial production. Flax seeds are rich in functional compounds with unique biological activities, including unsaturated fats, lignin, flax gum, and soluble dietary fiber. Flax seeds contain 35–45% oil, which consists of 5–6% palmitic acid (PA, C16:0, a saturated fatty acid with 16 carbon atoms and no double bonds), 3–6% stearic acid (SA, C18:0, a saturated fatty acid with 16 carbon atoms and no double bonds), 19–29% oleic acid (OA, C18:1, a monounsaturated fatty acid with 18 carbon atoms and one double bond), 14–18% linoleic acid (LA, C18:2, a polyunsaturated fatty acid with 18 carbon atoms and two double bonds), and over 50% α-linolenic acid (ALA, C18:3, a polyunsaturated fatty acid with 18 carbon atoms and three double bonds) [1]. OA is a vital fatty acid that is essential for both plant growth and human health. In humans, it plays an important role in repairing sun-damaged DNA [2], increasing the survival of neural stem cells [3], and reducing cardiovascular disease [4], arthritis [5], and aging [6]. In addition, seeds with high OA content are used in the production of biodiesel, providing a renewable and environmentally friendly alternative energy source [7]. The high OA content of flax not only increases its economic value but also strengthens the crop’s resistance and adaptability [8]. Conventional breeding techniques are limited by the narrow range of genetic variability, making it difficult to rapidly achieve high OA levels. As a result, mutation breeding and screening methods have emerged as an effective alternative.

In plants, fatty acid desaturases regulate the relative abundance of different fatty acid species and their derivatives [9,10]. SAD, also known as Δ9 desaturase, introduces the initial double bond between carbons 9 and 10 of saturated fatty acids. The enzyme plays a key role in the synthesis of monounsaturated fatty acids. Genes encoding SAD have been cloned from several plants including *Arabidopsis* [11], soybean [12], cotton [13], peanut [14], and rape [15]. The sequences of these genes are highly conserved, indicating that they have been subjected to strong evolutionary pressure. The expression level and enzyme activity of SAD regulate the ratio of saturated fatty acid to polyunsaturated fatty acids in plants [16]. In the cascade of fatty acid desaturation, following the action of SAD to generate monounsaturated fatty acids, FAD2 acts as a downstream key enzyme to further drive fatty acid unsaturation. FAD2, also known as ω-6 desaturase, catalyzes the further desaturation of C18 monounsaturated fatty acids, introducing double bonds at the Δ12 position of the acyl carbon chain. This key enzyme regulates the conversion of C18:1 to C18:2. Researchers have isolated and identified genes encoding the FAD2 protein in several crops, including sesame [17], maize [18], rape [19], olive [20], soybean [21], sunflower [22], and cotton [23]. This gene contains a conserved large intron in the 5’- untranslated region (5′ UTR), with the number of exons varying between different species. In addition, the copy number, expression pattern, and function of *FAD2* vary between species [24]. Previous studies have shown that *LuSAD* consists of two encoding genes, *LuSAD1* and *LuSAD2*. The expression of *LuSAD1* is more time-specific, with more pronounced differences between high and low ALA varieties. Conversely, *LuSAD2*, which comprises two subtypes, shows constitutive expression with a higher transcript level than *LuSAD1* [25]. The silencing of two *LuFAD2* genes in Linola, a high LA cultivar, resulted in OA accounting for 80% of the total fatty acid content [26].

In 1953, alkylating agents were discovered to be effective in inducing genetic mutations, thereby initiating the field of chemical mutagenesis which has been used to innovate crop germplasm and breeding [27]. In recent years, ethyl methyl sulfone (EMS) has been used as a means of increasing the oleic acid (OA). For example, in their study, Lee et al. used EMS mutagenesis to generate two mutant rapeseed (*Brassica napus* L.) lines with an average OA content of approximately 76% [28]. Using analogous methods, Fang et al. successfully generated a peanut mutant with an OA content of approximately 60%, probably due to alterations in the *FAD2B* gene [29]. Using EMS mutagenesis and several breeding generations, Tang et al. were able to increase the OA content of the *Brassica napus* cultivar “Zhong Shuang 11” by 4.78% [30]. In addition, Daurova et al. used EMS mutagenesis to generate diploid turnip rape (*Brassica rapa*) plants with an OA content of 11–12% [31]. In the aforementioned studies, the corresponding WT was used as the control, and the changes in fatty acids content of the mutants were based on comparisons with WT.

In this study, EMS mutagenesis was used to generate a flax mutant library, which was subsequently screened for mutants with high OA levels. The molecular mechanisms of a particularly high OA mutant, M45, were analyzed using BSA-Seq, RNA-Seq, and metabolomics, facilitating the identification of genes regulating OA content. This study provides a valuable germplasm resource for flax molecular breeding and a theoretical basis for further investigation into the molecular mechanisms of OA regulation.

## 2. Results

### 2.1. Construction of a Mutant Library and Investigation of Mutant Growth Phenotypes

**Figure 1:**
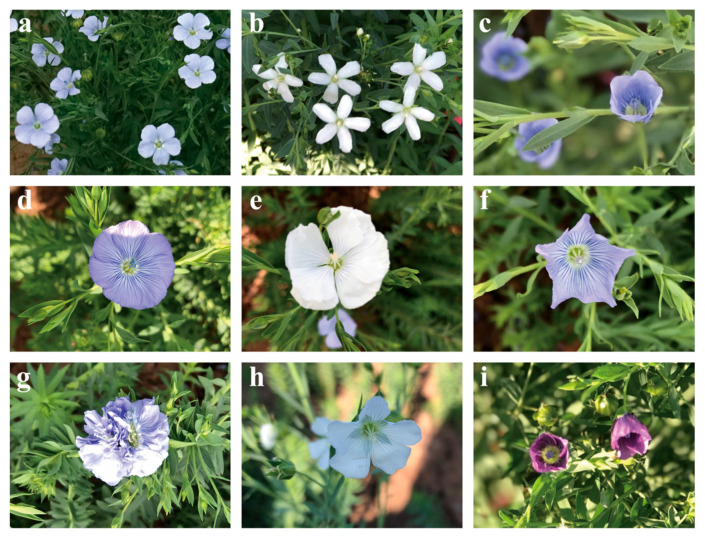
Flower mutant phenotypes in the M2 population: (**a**) WT with light purple flowers and 5 petals; (**b**) a mutant with white flowers and oval petals; (**c**) a mutant with petal aggregation; (**d**) a 7-petal mutant; (**e**) a mutant with eight petals and white flowers; (**f**) a floral variant mutant; (**g**) a mutant with wrinkled petals; (**h**) a mutant with blue-white flowers; (**i**) a mutant with dark purple flowers and petal aggregation.

**Figure 2:**
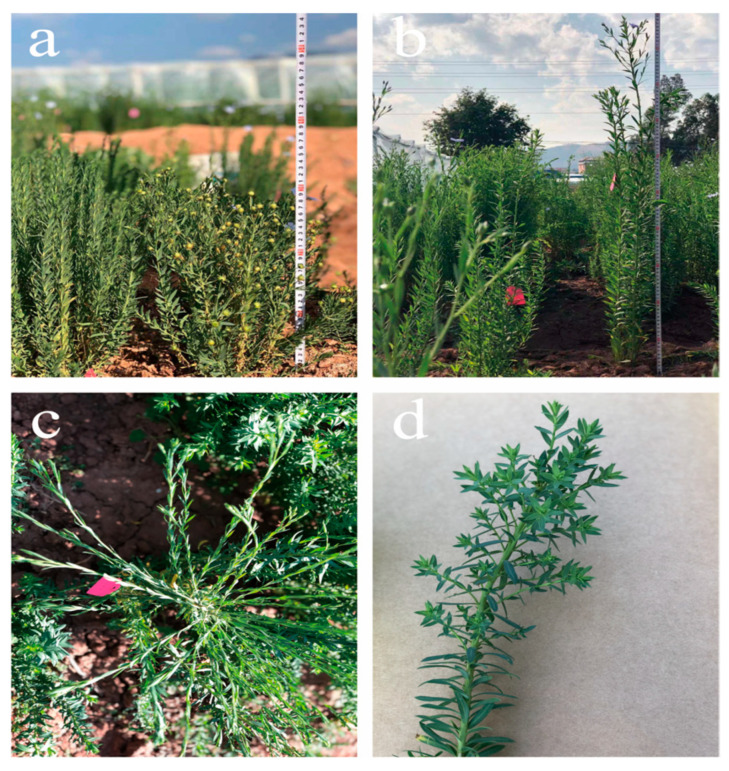
Typical plant mutant phenotypes in the M2 population: (**a**) a mutant with short pole; (**b**) a mutant with a tall pole; (**c**) a mutant with an umbrella-shaped main branch; (**d**) a mutant with increased secondary branching of primary branches. The tape measure used to measure plant height in the figure is marked in centimeters (cm).

Mutagenesis of WT flax seeds resulted in the generation of approximately 100,000 individuals in the M1 population. Subsequently, 5000 individuals were randomly harvested at maturity and screened for changes in oil content and fatty acids. The M2 population contained 4400 lines, of which 994 lines showed trait segregation. A field growth phenotype survey was conducted at the peak flowering stage, and 863 mutants with variable growth phenotypes were identified (Table 1). These included mutants with variation in flower type, flower color, and flowering time, such as a number of petals ranging from 4–8; flower color darkened to light purple or changed to white (Figure 1); mutants variation in plant type with the highest proportion, such as the main branch umbrella-shaped, creeping growth, or tall or short stem (Figure 2). In addition, mutants with smaller leaf pinching angles and thickened leaf blades were also observed.

### 2.2. Fatty Acid Mutants Screening

At the maturity stage of the M2 generation, 1185 plants capable of normal seed production were randomly selected for self-crossing to obtain the M3 generation, with no predetermined screening conditions applied, such as those relating to seed viability. There were 367 mutants with changes in fatty acid fractions screened, including 168 mutants with a 10% increase in oleic acid (OA) content, with a maximum of 42.3%; 51 mutants with a 3% decrease, with a minimum of 13.44%; 55 mutants with a 3% increase in linoleic acid (LA) content, with a maximum of 33.25%; 27 mutants with a 3% decrease, with a minimum of 7.59%; 116 mutants with a 4% increase in linoleic acid (ALA), with a maximum of 63.04%; and 136 mutants with a 10% decrease in linolenic acid, with a minimum of 32.07% (Table 2).

M4 seeds of each of the 367 mutant lines were mixed and harvested. The results showed that 26 mutants increased OA content by more than 5%, with a maximum of 37.38%; 16 mutants decreased OA content by more than 5%, with a minimum of 21.29%; 13 mutants increased LA content by more than 2.5%, with a maximum of 19.54%; 10 mutants decreased LA content by 1.5%, with a minimum of 8.13%; 29 mutants increased ALA content by more than 6%, with a maximum of 55.39%; 20 mutants decreased ALA content by 5%, with a minimum of 33.57% (Table 3). Later, 32 extreme mutants were screened from 108 M5 generation fatty acid mutants (Table S1).

Six of these lines were selected for cultivation in Lanzhou City, Gansu Province, China, the main flax production area. Among these lines, M45 had the highest relative OA content and was selected for further analysis (Table 4).

### 2.3. Fatty Acid Fractions Analysis

**Figure 3:**
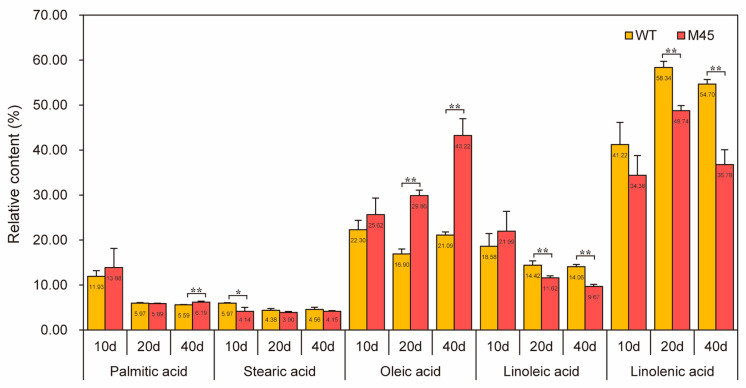
M45 and WT fatty acid fractions in three time periods. * represents *p* < 0.05; ** represents *p* < 0.01.

In order to understand the differences in fatty acid accumulation during the development of M45, a series of capsules were taken at 10, 20, and 40 days after flowering. These capsules were analyzed for their fatty acid composition, including the fractions of PA, SA, OA, LA, and ALA. On day 10, the percentage of SA in M45 was significantly lower than that of WT, at 4.14% compared to 5.97% (Figure 3). In addition, the percentage of ALA was observed to be lower than that of the WT, while PA, OA, and LAs were all higher than that of the WT, although none of these differences were found to be statistically significant. At day 20, no significant difference was observed between the PA and SA levels in the M45 and WT. The percentage of OA was significantly higher in M45 than in WT, with a difference of 12.96%. However, the levels of linoleic acid (11.62%) and linolenic acid (48.74%) were significantly lower than those of WT (14.42% and 58.34%, respectively). On day 40, there was no significant difference in SA content between M45 and WT. The percentages of linoleic and linolenic acids were significantly lower in M45 than in WT, with decreases of 4.39% and 17.92%, respectively. Conversely, the levels of PA and OA were significantly higher than in WT, with OA increasing by 21.23%. Overall, the trends of PA, SA, LA, and ALA in M45 and WT were consistent, with PA, SA, and LA continuing to decrease in the M45 mutant, while ALA initially increased and then decreased at day 20. In terms of OA accumulation, WT first decreased and then increased, but the mutant showed a steady increase to 43.22%. This final level was 14.17% higher than the average OA level of 116 local varieties from Gansu (China) [32]. The two genotypes showed contrasting patterns in terms of OA accumulation during the 10- to 20-day period, indicating that this stage is a critical period for changes in the fatty acid fractions of flax. This is in agreement with our previous findings [33,34]. Therefore, the 10-day and 20-day time points were selected for transcriptome and lipid metabolome analysis.

### 2.4. Lipid Metabolome Analysis

#### 2.4.1. Qualitative Lipid Results

**Figure 4:**
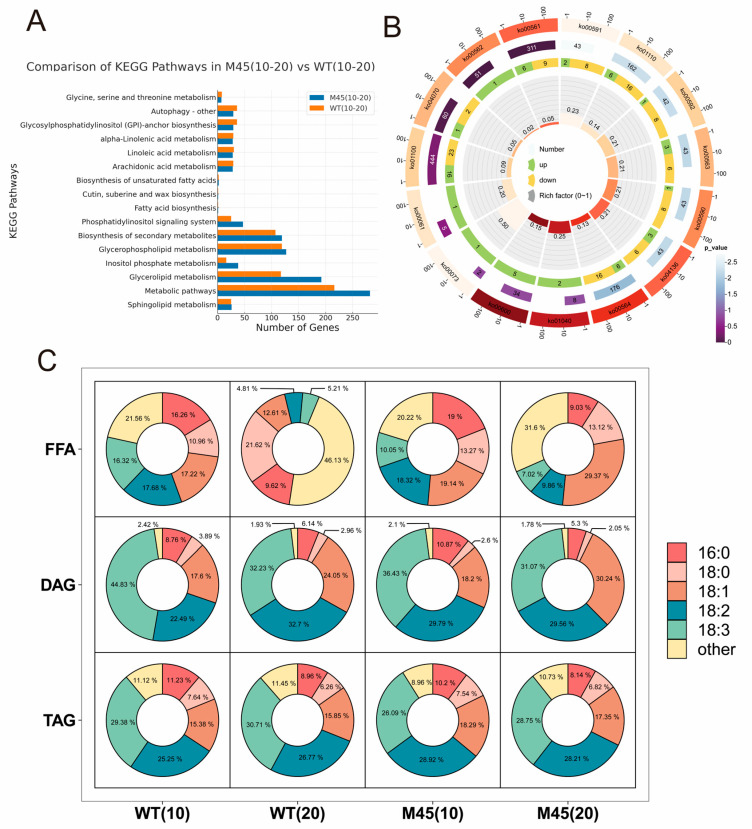
WT and M45 lipid metabolome analysis: (**A**) differential gene counts in the pathway for M45 and WT during the 10–20-day period of development; (**B**) the outermost circle (1, 10, 100) representing the gene number scale. The corresponding block is the pathway name and is used as the KEGG number. Different colors represent different KEGG pathways. One layer further inwards shows the total number of genes. The colors correspond to different *p* values. One layer further inwards shows the numbers of upregulated and downregulated genes. The yellow color indicates downregulation and the green color indicates upregulation. One layer further inwards shows the enrichment factor; (**C**) fatty acid composition of FFA, DAG, and TAG in M45 and WT at day 10 and day 20.

In order to clarify the differences in the metabolic mechanisms of M45 and WT oleic acid, lipid metabolome assays were performed on capsules harvested at 10 and 20 days after flowering. A total of 595 lipid compounds were identified in the four samples. The phenotypic results indicated that the accumulation of M45 and WT OA showed an inverse trend at day 10 compared to at day 20. Between 10 and 20 days of development, differences were observed between M45 and WT. A comparison of the lipid profiles of the WT at day 10 and day 20 revealed that 110 lipids were upregulated, and 211 lipids were downregulated at day 10 (Table S2). M45 showed an upregulation of 183 lipids and a downregulation of 192 lipids at this developmental stage. The KEGG classification showed that metabolic activity was more prevalent during the development of M45 (10–20 day) than WT. Metabolites related to glycerol ester metabolism (ko00561), phosphatidylinositol signaling system (ko04070), glycerophospholipid metabolism (ko00564), and phosphatidylinositol metabolism (ko00562) were found to be more active in M45. These pathways may be related to OA accumulation in M45 (Figure 4A). The difference in OA accumulation between WT and M45 after 20 days was statistically significant. The lipid characterization results showed that the M45 screening yielded 21 upregulated lipids compared to WT, mainly including 4 Cert (Phytoceramide), 3 PI (Phosphatidylinositol), 3 PE (Phosphatidylethanolamine), and 2 FFAs (Free fatty acids) (OA and LA); and 27 down-regulated lipids, mainly including 8 PC (Phosphatidylcholine), 7 TAG, and 6 PE (Table S2). The KEGG annotation results showed that the differential lipids were annotated into 15 pathways, mainly including LA metabolism (ko00591), secondary metabolite biosynthesis (ko01110), α-linolenic acid metabolism (ko00592), arachidonic acid metabolism (ko00590), glycerophospholipid metabolism (0ko00564), and unsaturated fatty acid biosynthesis (ko01040), among others (Figure 4B). The metabolomic data were consistent with the phenotypic results, thereby confirming the enrichment of numerous metabolites enriched in upstream and downstream pathways associated with OA synthesis.

#### 2.4.2. High OA Content in M45 Is Primarily Due to OA in FFA

The total lipid contents measured in the 10-day and 20-day capsules of M45 were 98,660.08 nmol/g and 193,138.78 nmol/g, respectively, which were approximately 34.72% and 13.51% lower than those in WT, respectively. The main forms of OA were glycerol esters and free oleic acid. The main constituents of linseed were triacylglycerols (TAG) and diacylglycerols (DAG). To elucidate the differences in fatty acid composition between M45 and WT lipids, we performed a structural analysis of the components of TAG, DAG, and FFA. The quantitative results showed that 42 different fatty acids were found in TAG, with PA, SA, oleic, LA, and ALA being the most abundant. In the M45 and WT samples harvested at day 10, the five aforementioned fatty acids accounted for 91.04% and 88.88%, respectively. Among them, ALA had the highest percentage (26.09%, 29.38%), followed by LA (28.92%, 25.25%) and OA (18.29%, 15.38%). Similar to the 10-day results, the 20-day samples showed that the percentage of the five fatty acid pairs remained above 88%. In addition, the percentages of linoleic and oleic acids were slightly higher in M45 than in WT (Figure 4C).

DAG, a pivotal intermediate in the synthesis of glycerol esters, was the second most prevalent compound in caraway samples, surpassed only by TAG. The analysis demonstrated that DAG contained 24 distinct fatty acids. The proportion of all five principal fatty acids exceeded 97.58%, a figure that surpassed that of TAG. In the 10-day samples, there was a notable divergence in the fatty acid composition between M45 and WT, with the most pronounced differences observed in LA and ALA. In comparison to the WT, the proportion of M45 LA was elevated by 7.30%, while the proportion of ALA was reduced by 8.4%. In the 20-day samples, compared with the 10-day samples, the percentage of OA in M45 and WT increased by 12.04% and 6.45%, respectively, while the percentage of ALA decreased by 5.36% and 12.60%, respectively. The difference was a 10.21% increase in the proportion of LA between M45 and WT (Figure 4C). Two genotypes exhibited principal differences in OA at day 20, with a 6.19% higher OA content in M45 compared to in WT. The total proportion of FFA in M45 was higher than that in WT, encompassing 20 distinct fatty acids. The five most abundant fatty acids accounted over 78.44% of the 10-day samples, with stearic acid (SA) and oleic acid (OA) exhibiting a slight elevation in M45 relative to in WT (2.31% and1.92%, respectively), while ALA exhibiting a slight decline in M45 (6.27%). The 20-day sample revealed that the percentage of five fatty acids in M45 was higher than that of WT. The OA of M45 exhibited an upward trend during the growth period, increasing from 19.14% at day 10 to 29.37% at day 20. In contrast, the WT exhibited a decreasing trend, which is in accordance with results on fatty acid composition gathered by gas chromatography analysis. Finally, the percentage of OA at day 20 was 19.75% higher in M45 than in WT (Figure 4C). The data indicate that FFAs followed by OA in DAG are the primary factors contributing to the elevated OA content observed in M45.

### 2.5. RNA-Seq Analysis

To investigate the molecular mechanisms of OA accumulation during seed development, transcriptome sequencing was performed on M45 and WT at days 10 and 20. The raw reads of all 12 samples exceeded 43.31 million, resulting in 40.53 million clean reads after quality control (error rate: < 0.03%; Q20: > 98.21%; Q30: > 94.55%; CG content: 49.05–52.05%) (Table S3). The proportion of reads that were successfully matched to the reference genome exceeded 95.80%, indicating a high level of quality. The results of the gene fluorescence quantification were found to be consistent with those of the transcriptomic analysis, thereby confirming the reliability of the data (Figure S1).

#### 2.5.1. Differential Analysis and KEGG Annotation

**Figure 5:**
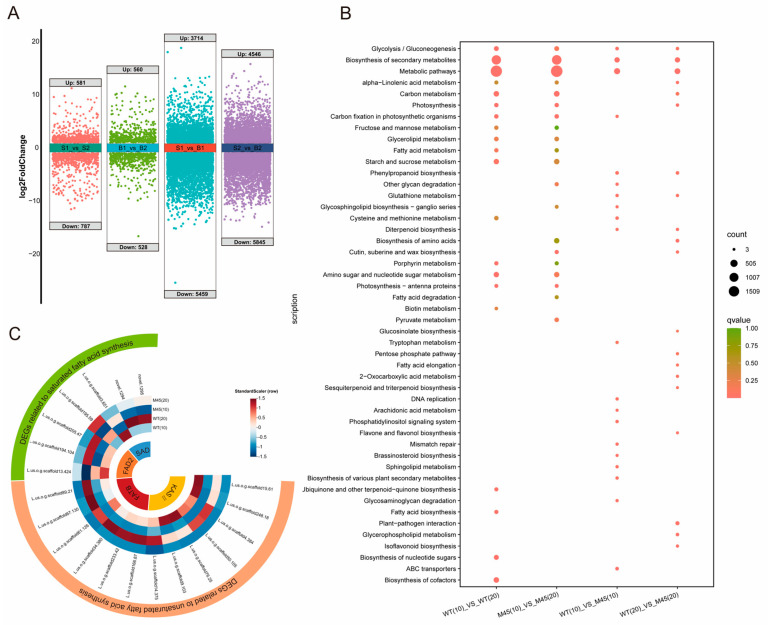
Analysis of M45 and WT RNA-Seq data: (**A**) differential gene volcano map of four differential combinations. S1 refers to WT(10), S2 refers to M45(10), B1 refers to WT(20), and B2 refers to M45(20); (**B**) KEGG functional enrichment of four combinations; (**C**) differential gene heatmap of two fatty acid synthesis pathways.

In the 10-day samples, 1368 differentially expressed genes (DEGs) were identified in M45 relative to WT, of which 581 were significantly upregulated and 787 were significantly downregulated. In the 20-day M45 samples, 1088 DEGs were present, of which 560 were significantly upregulated and 528 were significantly downregulated (Figure 5A) (Table S4). A KEGG analysis revealed that 19 pathways were significantly enriched (*p* < 0.05) for DEGs at day 10. These pathways were primarily related to metabolism (ko01100), secondary metabolite biosynthesis (ko01110), sphingolipid metabolism (ko00600), phosphatidylinositol signaling system (ko04070), arachidonic acid metabolism (ko00590), and diterpene biosynthesis (ko00904). The DEGs at day 20 revealed significant enrichment in 14 pathways, including ALA metabolism (ko00592), flavonoid biosynthesis (ko00943), glycerophospholipid metabolism (ko00564), and keratin, xylem, and waxy biosynthesis (ko00073) (Figure 5B). The transcriptome and metabolome data exhibit a consistent enrichment trend in pathways such as glycerophospholipid metabolism (ko00564), which collectively confirm the regulatory role of gene expression in lipid metabolism.

A comparison of the 10-day samples with the 20-day WT samples revealed the presence of 9173 differentially expressed genes (DEGs). Of these, 3714 were significantly upregulated, while 5459 were significantly downregulated. Concurrently, M45 exhibited a greater number of differentially expressed genes (DEGs), with 4546 significantly upregulated and 5845 significantly downregulated (Table S5). This indicates that these DEGs may play a role in seed development and fatty acid accumulation (Figure 5A). The KEGG analysis revealed that 36 and 40 pathways were significantly enriched pathways in WT and M45, respectively. A total of 32 common pathways were significantly enriched in both lines, with the pathways related to fatty acid biosynthesis (ko00061), cuticle, corky and waxy biosynthesis (ko00073), fatty acid elongation (ko00062), ALA metabolism (ko00592), glycerolipid metabolism (ko00561), and fatty acid metabolism (ko01212). This suggests that the anabolic reactions of lipids and fatty acids were active during the 10–20-day period. Additionally, the DEGs in M45 were significantly enriched in pathways related to fatty acid degradation (ko00071), arachidonic acid metabolism (ko00590), flavonoid biosynthesis (ko00941), and butyric acid metabolism (ko00650) (Figure 5B), indicating a potential correlation with the observed high OA traits.

#### 2.5.2. DEGs in Fatty Acid Synthesis

The study of the role of DEGs in fatty acid de novo synthesis provides insights into the regulatory mechanisms underlying alterations in fatty acid composition in mutant lines. The fatty acyl–acyl carrier protein thioesterase B (FATB) gene plays a pivotal role in the synthesis of fatty acids. It catalyzes the hydrolysis of palmitic-ACP and stearic-ACP, thereby converting them to long-chain saturated fatty acids such as PA and SA. Moreover, 3-keto-acyl carrier protein synthase II (KASII) is responsible for extending the carbon chain from C16 to C18. Both 6 KASII and 7 FATB were subjected to screening throughout the course of this study. The seven genes encoding *FATB* exhibited elevated expression levels in M45 relative to WT (at day 10), suggestive of a greater accumulation of PA and SA. Conversely, four of the six genes homologous to KASII exhibited reduced expression in M45 relative to WT, thereby facilitating the extension of PA to SA to occur at a greater extent in WT. In conclusion, at day 10, M45 exhibited elevated levels of PA and reduced levels of SA relative to WT. This observation may be attributed to differential expression of FATB and KAS II (Figure 5C).

#### 2.5.3. DEGs for Fatty Acid Synthesis

To elucidate the high OA properties of M45, we focused on enzymes related to the OA pathway during fatty acid desaturation. SAD is the rate-limiting enzyme for the desaturation of SA to OA, while *FAD2* is crucial for the further desaturation of OA to LA. In conjunction with the KEGG enrichment results, a total of 3 *SAD* homologous genes and 4 *FAD2* homologous genes were identified. The expression of these genes differed between M45 and WT at different developmental stages. Two *SAD* genes (*L.us.o.g.scaffold194.104*, *L.us.o.g.scaffold205.47*) showed higher expression levels in M45 than in WT, indicating that more SA was desaturated to produce OA. Conversely, the expression levels of *novel.1295* and *novel.1294*, which are homologous to *FAD2*, were found to be lower in M45 than in WT (at day 20), suggesting that they may be responsible for the desaturation of OA to LA during development (10–20 days) (Figure 5C).

### 2.6. Library Construction and BSA-Seq

#### 2.6.1. OA Content Variations

**Figure 6:**
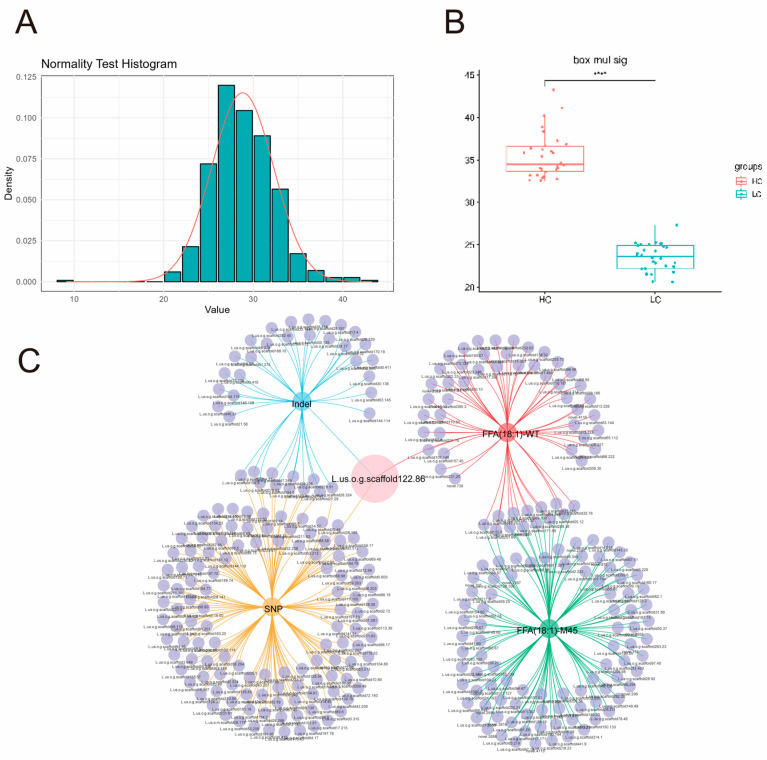
BSA-seq pool construction and combined analyses: (**A**) normal distribution test of oleic acid content in the F2 population; (**B**) OA content in extreme segregation pools; (**C**) BSA-seq, RNA-seq, and metabolomic association analyses: the blue color indicates genes associated with Indels, the gold color indicates genes associated with SNPs, the red color indicates DEGs associated with FFA (18:1) in WT (10–20 days), and the green color indicates DEGs associated with FFA (18:1) in M45 (10–20 days). The pink circle represents the screening candidate gene containing a mutation and associated with FFA (18:1).

To investigate the genetic loci associated with high OA in M45, an F_2_ segregating population was constructed by crossing M45 with WT. Analysis of the fatty acid fractions of 585 individuals from the F_2_ population revealed a continuous distribution of OA content. The high-content trait showed a normal distribution (Figure 6A) of a quantitatively inherited trait, which was consistent with the genetic model of high OA oilseed rape [35]. A total of 30 samples, containing both plants with high and low OA content, were selected from the F2 population. Each pool contained the extremes of OA content (Figure 6B).

#### 2.6.2. BSA-Seq Alignment

Whole genome resequencing was conducted on both the M45 and WT parents, in addition to the two segregation pools (HC and LC). A 350 bp WGS library was constructed, yielding 35.215 G of raw data, which was reduced to 34.433 G of clean data following quality control procedures (Q20: ≥ 95.91%; Q30: ≥ 89.43%; CG content of 38.27–38.42%). Sequence comparisons between the reads and the reference genome (306.4 Mb) were conducted using the BWA (Version: 0.7.10). The alignment rate across samples ranged from 96.65% to 97.18%, while the average depth of coverage (excluding the N region) ranged from 10.31 to 27.32X. The 1X and 4X coverage depths were consistently above 97.13% and 75.83%, respectively. These results indicated the high quality of the sequencing data, which enables the reliable detection of mutation sites (Table S3).

A total of 1,279,694 variant sites were identified, comprising 1,145,492 SNPs and 134,202 Indels. A differential analysis of the frequencies of SNPs and Indels in the offspring yielded 430,828 SNPs and 56,850 Indels. Of these, 640 SNPs and 119 Indels demonstrated statistically significant differences. The application of the ANNOVAR annotation facilitated the identification and selection of significant loci and genes exhibiting non-synonymous mutations. This resulted in 135 genes containing 180 trait-associated SNPs and 39 genes containing 42 trait-associated Indels (Table S5). It may be hypothesized that the mutated genes may be associated with the high OA observed in M45.

### 2.7. BSA-Seq, RNA-Seq, and Metabolome Analyses

To elucidate the correlation between differential genes and lipids, a correlation analysis was conducted on significant DEGs (Pearson correlation coefficient: >0.8), using 18:1 (Lipid-B-N-0028) in FFA as the target metabolite. In the WT and M45 (10–20 days) samples, the gene expression profiling yielded 57 and 102, respectively, that were significantly associated with FFA (18:1). A greater number of DEGs related to OA were observed in M45 than in WT. A total of 13 genes were identified as functioning in both WT and M45, suggesting the existence of significantly different mechanisms underlying OA accumulation. The shared genes were then compared with candidates from the BSA-seq, which revealed that only *L.us.o.g.scaffold122.86* exhibited both a regulatory function in OA and a variable locus. *L.us.o.g.scaffold122.86* displayed a negative correlation with OA accumulation. Transcriptome analysis revealed increased expression during development in WT but decreased expression in M45. These findings suggest that *L.us.o.g.scaffold122.86* may be the causal factor in the elevated OA accumulation observed in M45.

### 2.8. Regulatory Mechanisms of L.us.o.g.scaffold122.86

**Figure 7:**
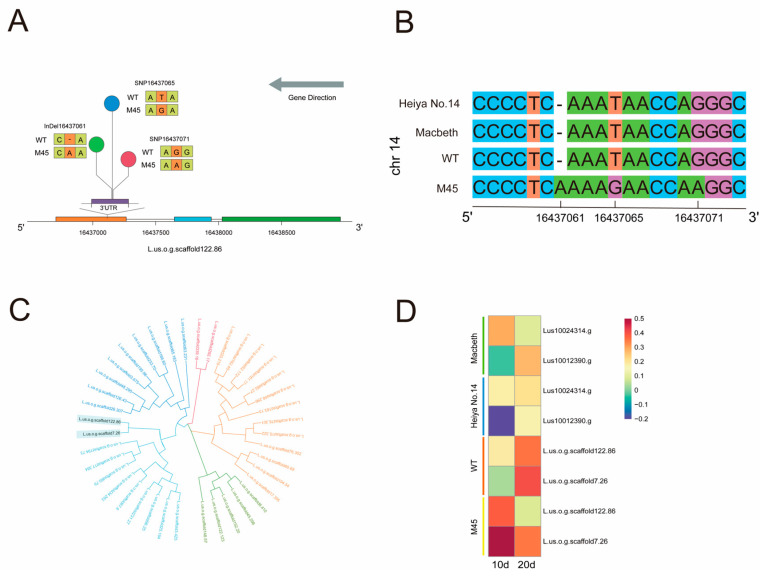
Analysis of *L.us.o.g.scaffold122.86* regulatory mechanisms: (**A**) mutation sites of *L.us.o.g.scaffold122.86*; (**B**) sequence alignment of *L.us.o.g.scaffold122.86* among flax varieties; (**C**) Evolutionary tree of significantly differentially expressed bZIP family genes in flax; (**D**) expression patterns of homologous genes between *L.us.o.g.scaffold122.86* and *L.us.o.g.scaffold7.26*.

The *L.us.o.g.scaffold122.86* sequence, located on chromosome 14, contained three base mutations in the 3′UTR: T→G (16,437,065), G→A (16,437,071), and an ala nine insertion (6,437,061) (Figure 7A). The sequence alignment results demonstrated that these three loci were present in M45, WT, and two additional flax varieties, Macbeth and Heiya No.14 (Figure 7B). *L.us.o.g.scaffold122.86* is a member of the basic leucine zipper (bZIP) transcription factor family, which plays a role in diverse regulatory processes among eukaryotic organisms. A protein sequence alignment revealed the highest degree of sequence similarity to *AtbZIP29* (*At4g38900*) from *Arabidopsis thaliana*. It is noteworthy that the two genes annotated as *FAD2* (*novel.1295* and *novel.1294*) in this study exhibited lower expression in M45 than in WT at day 20. These genes contribute to the accumulation of OA by preventing its desaturation into LA.

Given that genes within the same subfamily may exhibit analogous functions, we undertook an analysis of the bZIP family with the aim of identifying genes that interact with *L.us.o.g.scaffold122.86*. A total of 108 genes were identified through BLAST comparison, with 40 exhibiting significant differential expression. The phylogenetic tree revealed that *L.us.o.g.scaffold122.86* and *L.us.o.g.scaffold7.26* were grouped together in the same cluster (Figure 7C). Both genes exhibited comparable trends, with elevated expressions observed in WT but diminished expressions in M45 during the 10–20-day period. Furthermore, these genes were identified in Macbeth and Heiya No.14 [34]. A BLAST analysis of the transcriptome data identified two highly homologous genes, *Lus10024314.g* and *Lus10012390.g*), which exhibited > 90% similarity to *L.us.o.g.scaffold122.86* and *L.us.o.g.scaffold7.26*, respectively. In Heiya No.14, which has been bred for its fiber content, the expression pattern of these two homologous genes reflected that of WT and was opposite to that observed in M45. In Macbeth, bred for oil content, the expression pattern of *Lus10012390.g* was found to be consistent with that of WT, whereas the expression trend of *Lus10024314.g* matched that of M45 (Figure 7D). It is hypothesized that this may be attributed to varietal differences. These findings demonstrate that the differential expression of *L.us.o.g.scaffold122.86* and *L.us.o.g.scaffold7.26* may be associated with high OA content.

## 3. Discussion

In this study, EMS mutagenesis was employed to construct a flax mutant library. The phenotypic differences in flowers, stems, and branches of mutants obtained via EMS mutagenesis validate the effectiveness of mutagenesis and reflect the phenotypic diversity of the mutant library. M45 was identified as a mutant with an OA content 21.23% greater than that of the WT. During the 10–20-day capsule development period, M45 displayed substantial discrepancies in OA accumulation comparing with WT. These differences were primarily attributed to free fatty acids. Analyses of the lipid metabolome and RNA-Seq data revealed that the observed differences between M45 and WT were consistent with their respective phenotypes. A total of 222 OA-related variable sites were identified through BSA-Seq and integrated with RNA-Seq and metabolomic data to construct an association network. The mutant gene *L.us.o.g.scaffold122.86*, which has been annotated as a bZIP transcription factor, was screened for mutations associated with OA. Gene family and functional analyses suggested that the gene may work with *L.us.o.g.scaffold7.26* to affect OA accumulation.

In plants, bZIPs have been demonstrated to play a pivotal role in the regulation of diverse physiological processes, including pathogen defense, light and stress signaling, seed maturation, and flower development [36]. Despite the absence of experimental validation of a direct association between bZIP factors and OA accumulation, conclusions regarding the regulation of fatty acid metabolism by the bZIP family have been reported in other studies. For instance, in the fungus Beauveria bassiana, the bZIP transcription factor HapX (BbHapX) has been demonstrated to regulate fatty acid metabolism in conidia, with *Ole1* (a Δ9-fatty acid desaturase gene) confirmed as a downstream target of HapX [37]. This finding suggests that the regulatory mechanism of fatty acid desaturase genes by bZIP factors displays a degree of conservation across diverse organisms. Analogous regulatory relationships have been identified in the plant kingdom. As stated in Reference 3, bZIP67 is regarded as a master regulator of fatty acid storage; increased levels of bZIP67 result in elevated expression of the FAD3 desaturase, which catalyzes the conversion of linoleic acid to linolenic acid [38]. This finding suggests that the regulation of desaturase gene expression by the bZIP family members is likely to be a prevalent feature in the metabolic pathways of plant fatty acids.

It is hypothesized that mutations in *L.us.o.g.scaffold122.86* are responsible for the observed changes in OA accumulation. This study provides novel materials and insights that will enhance our understanding of the regulatory pathways in plant fatty acid metabolism.

## 4. Materials and Methods

### 4.1. Plant Materials and Determination of Fatty Acid Content

The flax variety Longya 10 was selected as the WT for this study. A total of 5 kg seeds were soaked in distilled water for 10 h and then drained. The seeds were then added to a 0.9% EMS solution in a fume hood and placed on a shaker for 18 h to allow full contact with the mutagenic solution. The seeds were then placed in mesh bags, rinsed with running water for six more hours, dried with filter paper and designated as “M1” seeds. The seeds were grown, self-pollinated and screened for several generations in Yuanmou City (Yunnan Province, China), Langfang City (Hebei Province, China), and Lanzhou City (Gansu Province, China). The capsules were collected from M45 and WT at 10, 20, and 40 days after flowering, frozen in liquid nitrogen and stored at −80 °C for subsequent experiments [34]. The fatty acid composition of seeds was determined using an infrared analyzer DA7200 (Perten Instruments Co., Ltd., Stockholm, Sweden). At each specified time point, the analysis was repeated four times, with the mean value taken as the final content. For the fatty acid composition of capsules at 10, 20, and 40 days, gas chromatography was employed. At each designated time point, the analysis was performed in triplicate, and the mean value was used as the final content.

### 4.2. Determination of Plant Lipids

Quantitative lipid metabolome analysis was performed on the capsules of M45 and Longya 10 at 10 days and 20 days after flowering conducted by Metware (Metware, Wuhan, China). Qualitative and quantitative detection of lipids was carried out using ultra-performance liquid chromatography (UPLC, ExionLC™ AD) coupled with tandem mass spectrometry (MS/MS, QTRAP^®^ 6500+), with a Thermo Accucore™ C30 liquid chromatography column. The sample processing method was as follows: Weigh 20 mg of a freeze-dried sample into a 2 mL centrifuge tube containing steel beads, add 1 mL of a lipid extraction solution with the internal standard (methyl tert-butyl ether:methanol ratio = 3:1, *v*/*v*), and shake for 30 min. Add 300 μL of ultrapure water, shake for 1 min, and let the mixture stand at 4 °C for 10 min. Centrifuge at 12,000 rpm at 4 °C for 3 min, transfer 400 μL of the supernatant to a 1.5 mL centrifuge tube, and concentrate to complete dryness at 20 °C (approximately 2 h). Add 200 μL of a lipid reconstitution solution (acetonitrile:isopropanol ratio = 1:1, *v*/*v*), vortex for 3 min to reconstitute, and centrifuge at 12,000 rpm at 4 °C for 10 min. Transfer 120 μL of the supernatant into the glass insert of the sample vial for LC-MS/MS detection. Statistical tests were performed in R using principal component analysis, hierarchical cluster analysis, and Pearson product-moment correlation coefficient calculation. Differences between groups were considered significant if the fold change was greater than 2 or less than 0.5. Significance of the pathways was determined by the *p*-values from hypergeometric tests.

### 4.3. RNA-Seq and Real-Time Quantitative PCR of High OA Mutants

Transcriptome sequencing was performed on total RNA from the capsules collected at 10–20 days after flowering. Libraries were prepared using the NEBNext^®^ Ultra™ RNA Library Prep Kit (Illumina, USA). Samples were sequenced using the Illumina platform, with three biological replicates for each time point. Raw reads were filtered using FASTP and aligned to the reference genome ASM1066527V2 (FTP: ftp.ncbi.nlm.nih.gov/genomes/AL665275GCA) using HISAT2 [39]. Gene quantification was performed using feature counts. The false discovery rate (FDR) was obtained using DESEQ2 [40] for differential expression analysis, and *p*-value correction was performed using the Benjamini−Hochberg method. The threshold for significant differential expression was defined as a log2 fold change of at least 1 and a false discovery rate (FDR) of less than 0.05. To verify the accuracy of the transcriptome data, specific primers were designed (Table S6), and *LuACTIN* was used as an endogenous reference gene. Three technical replicates and three biological replicates were used for each treatment.

### 4.4. Construction of High OA Populations and BSA-Seq

The stable M45 mutant was crossed with WT to obtain F_1_ and F_2_ segregating populations. The F_2_ population was sown in Lanzhou City (36°19′55.78″ N, 103°56′51.04″ E). A total of 600 F_2_ plants were harvested individually at the stage of maturity. The remaining F_2_ plants were then mixed and harvested. Samples were taken from 30 high-OA (HP) F_2_ plants and 30 low-OA (LP) content F_2_ plants, and DNA was extracted using the SDS method.

DNA samples were randomly fragmented into 350 bp fragments using a Covaris Crusher LE220R-plus (Covaris, Woburn, MA, USA), and DNA libraries were constructed in accordance with the TruSeq Library Construction Kit (Illumina, USA). Samples were sequenced using the Illumina HISEQTMPE150 sequencing platform (Beijing Nuohe Zhiyuan Science and Technology Co., Ltd., Beijing, China). Raw reads were filtered to remove those containing linkers, an excess of unknown nucleotides greater than 10%, or a high proportion of low-quality base calls (Q ≤ 5). The clean reads were then aligned to the flax reference genome using BWA [41]. SNPs and Indels were identified using GATK [42], filtered by variant filtration and annotated using ANNOVAR (Version: 2013Aug23). ByM45 was selected as the reference parent based on SNP/Indel-index mapping, and markers for homozygous differences between the two parents were screened for an SNP/Indel index of < 0.3 or a depth of < 7 and SNP/Indel index deletion in the offspring. To ensure that minor quantitative trait loci (QTLs) were not missed, SNP/Indels with significant differences in SNP/Indel index between HP and LP loci across the genome were selected. Significant differences were defined as those with an NP/Indel index of 0.8 in the LP progeny and an SNP/Indel index of ≤ 0.2 in the HP progeny. The results of the ANNOVAR annotation were extracted, and the following categories of genes were identified as potential candidates: those located upstream, those involved in stop loss or gain, those encoding non-synonymous mutations, and those involved in alternative splicing.

### 4.5. Data Processing

Sequences were aligned and a gene family phylogenetic tree was constructed using MAGE11. A normality test and Pearson correlation analysis were performed using SPSSPRO (https://www.SPSSPRO.com/ (accessed on 3 September 2022)) to examine the relationship between OA content and fatty acid composition in all samples of the F_2_ population. The data presented are the mean and standard deviation (SD) of the three replicate analyses. A two-tailed *t*-test was used for post hoc analyses.

## Figures and Tables

**Table 1:** Statistics of mutants related to plant type, flower type and color, florescence, and leaf shape in the M2 population.

Phenotypic Category	Phenotype	Number
plant type	prostrate growth, increased lateral branching	63
	increased secondary branching of primary branches	58
	umbrella-shaped main branch	120
	short pole	102
	tall pole	25
	slender stem	157
	thick stem	54
	culms flattened, leafy growth	8
flower type and color	4-petal flower	3
	6-petal flower	88
	7-petal flower	17
	8-petal flower	3
	petal wrinkling	15
	small flower size	13
	large flower size	8
	dark color	11
	light color	5
	white flower	21
florescence	early flowering	371
	early flowering	122
leaf shape	small leaf angle	15
	thickening of leaf blades	11

**Table 2:** Summary of fatty acid mutants in 1185 M3 seeds.

Fatty Acid	Filter Criteria (%)	Number	Content in WT (%)	Area
OA	≥30.58	168	20.58 ± 3.12	Yuanmou (China)
	≤17.58	51		
LA	≥17.95	55	14.95 ± 0.59	Yuanmou (China)
	≤11.95	27		
ALA	≥56.57	116	52.57 ± 2.64	Yuanmou (China)
	≤42.57	136		

Note: Data represent the mean and standard deviation (STD) of three replicates.

**Table 3:** Summary of fatty acid mutants in 367 M4 seeds.

Fatty Acid	Filter Criteria (%)	Number	Content in WT (%)	Area
OA	≥34.72	26	29.72 ± 0.91	Langfang (China)
	≤24.72	16		
LA	≥13.17	13	10.67 ± 0.05	Langfang (China)
	≤9.17	10		
ALA	≥50.91	29	44.91 ± 0.89	Langfang (China)
	≤39.91	20		

Note: Data represent the mean and standard deviation (STD) of three replicates.

**Table 4:** Summary of M6 seed fatty acid extremity mutants.

Phenotypic Category	Name	OA (%)	Percentage Increase	ALA (%)	Percentage Increase
-	WT	29.78	-	45.43	-
OA	M45	37.23	7.45	36.80	-
	M60	34.02	4.24	40.17	-
ALA	M730	25.53	-	52.94	7.51
	M930	25.74	-	51.12	5.69
	M350	26.28	-	50.72	5.29
	M295	25.98	-	50.58	5.15
	M415	26.57	-	50.52	5.09

## Data Availability

The transcriptome raw data of M45 and WT (Longya No.10) were obtained in the NCBI database under the project number PRJNA883991 (https://www.ncbi.nlm.nih.gov/bioproject/PRJNA883991) (accessed on 24 September 2022).

## Supplementary Materials

The following supporting information can be downloaded at: https://www.mdpi.com/article/10.3390/plants14162583/s1.
